# Ocean acidification affects the expression of neuroplasticity and neuromodulation markers in seabream

**DOI:** 10.1242/bio.059073

**Published:** 2022-03-18

**Authors:** Rita A. Costa, Aurora Olvera, Deborah M. Power, Zélia Velez

**Affiliations:** Comparative Endocrinology and Integrative Biology Group, Centre for Marine Sciences, University of Algarve, Campus of Gambelas, Building 7, 8005-139 Faro, Portugal

**Keywords:** Ocean acidification, Gilthead seabream, Olfactory epithelium, Thyroid hormones, Neuroplasticity, Neuromodulation

## Abstract

A possible explanation for acidification-induced changes in fish behaviour is that acidification interferes with neurogenesis and modifies the plasticity of neuronal circuitry in the brain. We tested the effects on the olfactory system and brain of gilthead seabream (*Sparus aurata*) to 4 weeks' exposure to ocean acidification (OA). Olfactory epithelium (OE) morphology changed shortly after OA exposure and persisted over the 4 weeks. Expression of genes related to olfactory transduction, neuronal excitability, synaptic plasticity, GABAergic innervation, and cell proliferation were unchanged in the OE and olfactory bulb (OB) after 4 weeks' exposure. Short-term changes in the ionic content of plasma and extradural fluid (EDF) returned to control levels after 4 weeks' exposure, except for [Cl^−^], which remained elevated. This suggests that, in general, there is an early physiological response to OA and by 4 weeks a new homeostatic status is achieved. However, expression of genes involved in proliferation, differentiation and survival of undifferentiated neurons were modified in the brain. In the same brain areas, expression of thyroid hormone signalling genes was altered suggesting modifications in the thyroid-system may be linked to the changes in neuroplasticity and neurogenesis. Overall, the results of the current study are consistent with and effect of OA on neuroplasticity.

## Abstract (precis)

**Summary:** Ocean acidification alters fish behaviour. We show altered expression of genes involved in neuroplasticity and neuromodulation in fish exposed to high *P*CO_2_, highlighting their possible roles in such behavioural alterations.

## INTRODUCTION

Since the beginning of the industrial revolution, atmospheric carbon dioxide (CO_2_) has increased from 280 ppm to present day values (∼400 ppm). Roughly one-third of the CO_2_ released into the atmosphere by human activities has been absorbed by the ocean; when CO_2_ dissolves in seawater the pH decreases, a phenomenon termed ‘ocean acidification’ (OA). Global mean oceanic CO_2_ values are currently around 400 µatm and are expected to reach 1000 µatm by the year 2100, and 1900 µatm CO_2_ by 2300 (Caldeira and Wickett, 2003), levels that represent a potential threat to organisms and ecosystems (Doney et al., 2012). Laboratory experiments have shown that marine fish in water with a CO_2_ partial pressure (*P*CO_2_) equivalent to values predicted for the end of this century change their physiology, including neurosensory and behavioural systems (Dixson et al., 2010; Heuer and Grosell, 2014). Although controversial, the impact of OA on fish behaviour (Clark et al., 2020; Munday et al., 2020) has been linked to modified neurotransmitter function due to a shift in acid-base regulation caused by changes in extracellular ion concentrations (Nilsson et al., 2012). It has been proposed that increased water *P*CO_2_ alters blood and tissue levels of extracellular Cl^−^ and HCO_3_^−^ ions and their distribution over neuronal membranes, and this causes the GABAergic system of the central nervous system (CNS) to switch function from inhibitory to excitatory (the GABA_A_ theory) (Nilsson et al., 2012). Furthermore, during early development and adulthood, in immature neurons, GABA_A_ receptor activation is depolarizing and mainly excitatory (LoTurco et al., 1995; Wang and Kriegstein, 2009). Evidence supporting the GABA_A_ theory are the reported capacity of gabazine to reverse OA driven behavioural alteration (Chivers et al., 2014; Chung et al., 2014; Hamilton et al., 2014; Nilsson et al., 2012), exposure of spiny damselfish to high CO_2_ induces an increase in extra and intracellular HCO_3_^−^ concentrations, higher intracellular pH (Heuer et al., 2016), and overexpression of genes related to GABAergic neurotransmission (Schunter et al., 2018). However, another alternative and/or complementary explanation to OA driven behaviour alterations may be that increased *P*CO_2_ and/or decreased pH directly affect olfactory perception and the associated behaviour. Electrophysiological studies reveal that CO_2_-acidified seawater causes an immediate and reversible reduction in olfactory sensitivity to some odorants in the gilthead seabream (Velez et al., 2019), sea bass (Porteus et al., 2018) and crabs (Roggatz et al., 2016). This can be explained, in part, by conformational changes in the odorant and/or binding domain of the receptor (due to increased protonation), which reduces receptor-ligand binding affinity (Velez et al., 2019). Another possible explanation for fish behavioural alterations due to increased water *P*CO_2_, hitherto almost unexplored, is that OA may interfere with neurogenesis and modify the plasticity of brain neuronal circuitry. Teleosts experiencing challenging environments adjust their physiology and behaviour as part of their coping response, a phenomenon underpinned by neural plasticity (Ebbesson and Braithwaite, 2012). Studies with the three-spined stickleback (*Gasterosteus aculeatus*) revealed that exposure to elevated CO_2_ caused increased transcription of factors involved in neurogenesis, suggesting that exposure to low pH/high *P*CO_2_ water triggers a coping response resulting from neurophysiological plasticity. However, cinnamon anemonefish (*Amphiprion melanopus*) and spiny damselfish (*Acanthochromis polyacanthus*) exposed to the same conditions did not respond in the same way, suggesting that regulation of genes involved in neurogenesis in response to OA may be species-specific (Lai et al., 2017b).

In contrast to most vertebrates in which adult neurogenesis is highly restricted to certain areas of the CNS, in teleosts neurogenesis is not restricted and is lifelong (Zupanc and Sîrbulescu, 2011). Based on the environmental effects on fish brain plasticity and neurogenesis (Ebbesson and Braithwaite, 2012) we hypothesised that neuroplasticity and neurogenesis may occur as a physiological response to OA. In both vertebrates and invertebrates, adult neurogenesis is regulated by hormones, neurotransmitters, growth factors and environmental cues (Cayre et al., 2002). Moreover, it is well established that the CNS is highly sensitive to environmental changes, and the thyroid hormones (THs) are among the most important messengers regulating CNS maintenance throughout the entire lifespan of vertebrates. In addition, it is known that changes in water pH affects fish endocrine physiology, altering the thyroid system. Although scarcely analysed, it has been reported that exposure to acidic water increases T4 plasma levels in the climbing perch (*Anabas testudineus*) (Subhash Peter and Rejitha, 2011) and brown trout (*Salmo trutta*) (Brown et al., 1989), and decreases T3 levels in Atlantic Salmon (*Salmo salar*) (Brown et al., 1990). THs influence neuromotor performance, mood, memory, and cognition through the regulation of neurogenesis and proliferation (Bernal, 2017), prompting the hypothesis that OA may induce thyroid axis disruption, which in turn, may cause neuronal and consequent behavioural disturbances. To evaluate whether exposure to future *P*CO_2_ conditions could alter the ionic content of brain fluids and the thyroid axis and change neuromodulation and neuroplasticity, seabream was exposed for 4 weeks to low pH/high *P*CO_2_ water. This work was carried out using the seabream as it is a valuable aquaculture species in southern Europe, and therefore easy to obtain and maintain; furthermore, the genome has been sequenced (Pauletto et al., 2018), making primer design easier. During exposure, the ionic homeostasis of fish was monitored by assessing plasma and extradural fluid (EDF) ion composition. The expression of transcripts related to (i) neurogenesis and neuronal differentiation (*bdnf*) and (ii) TH signalling genes (*thra*, *thrb*) and deiodinase type 2 (*dio2*) was evaluated in the telencephalon, pituitary and thalamus. To assess general effects of OA on TH metabolism, liver expression of TH related genes was also measured. In addition, the occurrence of changes in olfactory perception were assessed by quantifying transcripts encoding proteins involved in (i) olfactory receptor cell activation (*g-olf*), (ii) neuronal excitability (*cacna2d*, *kcnn3*), (iii) synaptic plasticity (*gria4a*, *grm4*), (iv) GABAergic innervation (*gabra1*, *gabbr2*) and (v) cell proliferation (*pcna*). To evaluate whether morphological changes in the olfactory epithelium (OE) were coupled to putative changes in gene expression, histological analysis of the OE was carried out on a weekly basis.

## RESULTS

### Variation in the ionic content of plasma and EDF due to OA exposure

**Fig. 1.**
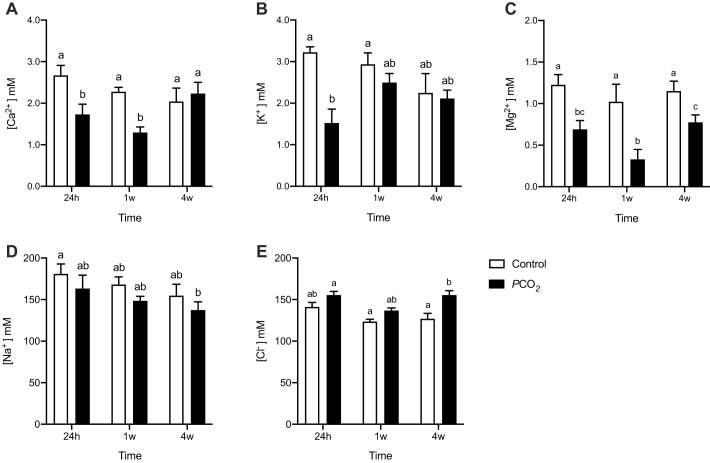
**Plasma ions concentration in seabream control fish and fish exposed to low pH/high *P*CO_2_ seawater for 24 h, 1 week and 4 weeks.** (A) Ca^2+^, (B) K^+^, (C) Mg^2^, (D) Na^+^ and (E) Cl^−^. Groups that are significantly different (two-way ANOVA followed by a Tukey multiple comparisons test, *P*<0.05) are indicated by different letters. The results are shown as the mean+s.e.m.; *n*=6.

The plasma concentration of Ca^2+^ (Fig. 1A) was significantly lower in fish kept in low pH/high *P*CO_2_ for 24 h (1.73±0.17 mM) and 1 week (1.35±0.10 mM) than in control fish (2.67±0.16 and 2.27±0.07 mM, respectively).

Differences between the Ca^2+^ concentration of control and treated fish after 24 h and after 1 week of exposure were higher, and not significantly different (*P*=0.9572) after 4 weeks of OA exposure. The concentration of K^+^ (Fig. 1B) was significantly lower in fish kept in low pH/high *P*CO_2_ for 24 h (*P*=0.0035) but not in fish exposed to these conditions for 1 week (*P*=0.91) or 4 weeks (*P*=0.99). The concentration of Mg^2+^ (Fig. 1C) was significantly lower in fish kept in low pH/high *P*CO_2_ for 24 h (*P*=0.0012), 1 week (*P*<0.0001) and 4 weeks (*P*=0.049) when compared to control fish. The concentration of plasma Na^+^ (Fig. 1D) was not significantly different between control and treated fish at any timepoint. The concentration of plasma Cl^−^ (Fig. 1E) did not vary between control and treated fish after 24 h (*P*=0.30) and 1 week exposure (*P*=0.39) but after 4 weeks it was significantly higher (*P*=0.0027) in low pH/high *P*CO_2_ fish.

**Fig. 2.**
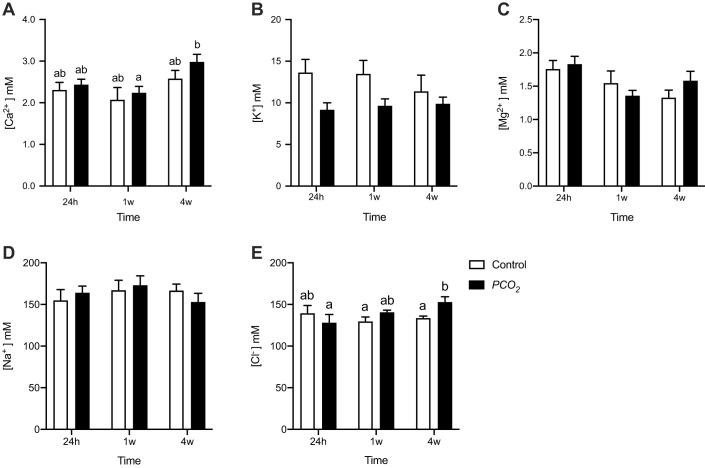
**Concentration of ions in the extradural fluid (EDF) of seabream in control fish and fish exposed to low pH/high *P*CO_2_ seawater for 24 h, 1 week and 4 weeks.** (A) Ca^2+^, (B) K^+^, (C) Mg^2+^, (D) Na^+^ and (E) Cl^−^. Significant differences were detected with two-way ANOVA followed by a Tukey multiple comparisons test (*P*<0.05) and are indicated by different letters above the bars in the graphs. Values are shown as the mean+s.e.m.; *n*=6.

The concentration of the ions, K^+^, Mg^2+^ and Na^+^ in the EDF (Fig. 2) of control fish and low pH/high *P*CO_2_ exposed fish was not significantly different at any time tested (Fig. 2A–D). The concentration of Ca^2+^ in fish exposed to low pH/high *P*CO_2_ for 4 weeks significantly increased (*P*=0.03) from 1 week to 4 weeks of exposure. The concentration of Cl^−^ (Fig. 2E) did not change in control and treated fish after 24 h (*P*=0.504) and 1 week of exposure (*P*=0.412) but after 4 weeks it was significantly higher (*P*=0.049) in fish experiencing low pH/high *P*CO_2_ conditions (Fig. 2E).

### Histomorphometry of the OE of seabream

**Fig. 3.**
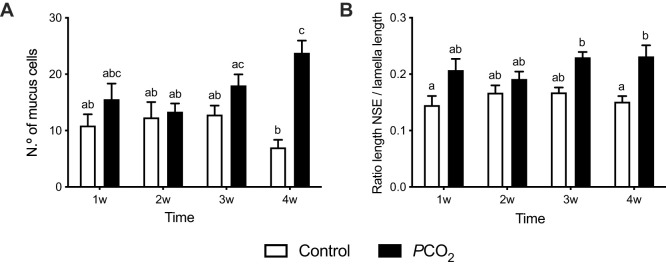
**Histomorphometry of the olfactory epithelium of seabream during exposure to low pH/high *P*CO_2_ seawater.** (A) Number of mucus cells and (B) Ratio of the NSE length/lamella length. The results are presented as mean+s.e.m. (*n*=3/ group) and were compared using a two-way ANOVA (*P*<0.05). Statistically significant differences between the low pH/high *P*CO_2_ and control group across time are indicated by different letters in the graphs.

The histomorphometric analysis of the OE of seabream (Fig. 3) revealed morphological alterations induced by the low pH/high *P*CO_2_ conditions. The number of mucus cells (Fig. 3A) in the non-sensory epithelium progressively increased across time in the low pH/high *P*CO_2_ exposed fish compared to the control. After 4 weeks of exposure, the number of mucous cells was significantly higher (*P*<0.0001) in the treated fish (23.8±2.2 mucus cells) compared to the control group (7.0±1.4 mucus cells). The length of the sensory epithelium was determined indirectly by the ratio of the non-sensory epithelium (NSE) length/lamella length (Fig. 3B) and progressively increased (*P*<0.05) across time in the low pH/high *P*CO_2_ exposed fish compared to the control fish, which did not change (*P*>0.05). After 4 weeks of exposure, the NSE/lamella ratio was significantly increased (*P*=0.02) in the low pH/high *P*CO_2_-treated fish compared to the control group, which represents a reduction in the contribution of the sensory epithelium to the total area of the olfactory epithelium.

### Gene expression under OA conditions

#### Olfactory rosette and olfactory bulb of seabream

**Fig. 4.**
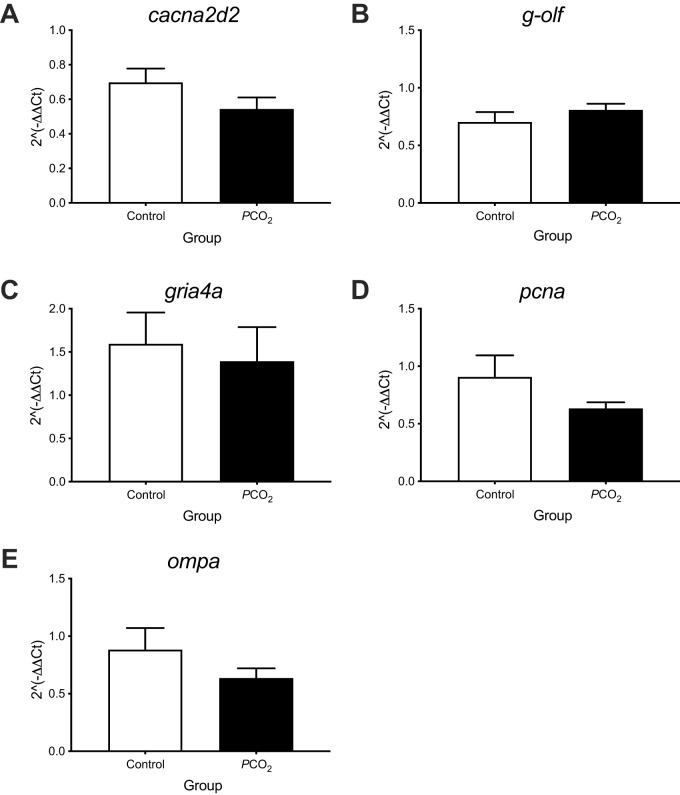
**Gene expression in the olfactory rosettes of seabream after the 4 weeks’ exposure to control (*n*=6) or low pH/high *P*CO_2_ (*n*=5-6) seawater conditions.** (A) *cacna2d*, (B) *g-olf*, (C) *gria4a*, (D) *pcna* and (E) *ompa*. Statistical significance was assessed using a Student′s *t*-test (*P*<0.05). No significant differences were found in target genes between the control and low pH/high *P*CO_2_ exposed fish. The results are shown as the mean+s.e.m.

In the OE, the expression of five different transcripts, *cacna2d*, *g-olf*, *gria4a*, *ompa* and *pcna*, was analysed after 4 weeks of exposure to low pH/high *P*CO_2_. No significant differences (*P*>0.05) were detected in the abundance of any of the gene transcripts in the olfactory rosettes between control and low pH/high *P*CO_2_-exposed fish after 4 weeks (Fig. 4).

**Fig. 5.**
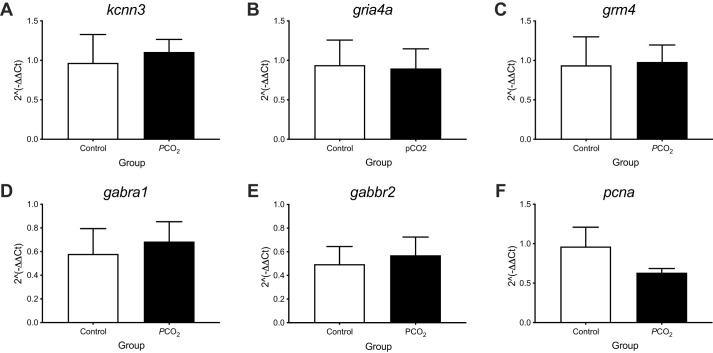
**Gene expression in the olfactory bulb of seabream after the 4 weeks’ exposure to control (*n*=4) or low pH/high *P*CO_2_ (*n*=3-4) seawater conditions.** (A) *kcnn3*, (B) *gria4a*, (C) *grm4*, (D) *gabra1*, (E) *gabbr2* and (F) *pcna*. Statistical significance was assessed using a Student′s *t*-test (*P*<0.05). No significant differences were found between the control and treatment group. The results are shown as the mean+s.e.m.

The expression of *kcnn3*, *gria4a*, *grm4*, *gabra1*, *gabbr2* and *pcna* was measured by RT-qPCR in the OB of seabream exposed for 4 weeks to low pH/high *P*CO_2_ or control conditions (Fig. 5)*.* No significant differences (*P*>0.05) in gene transcript abundance in the olfactory bulb were detected between control and treated fish.

#### Telencephalon, thalamus, pituitary and liver

**Fig. 6.**
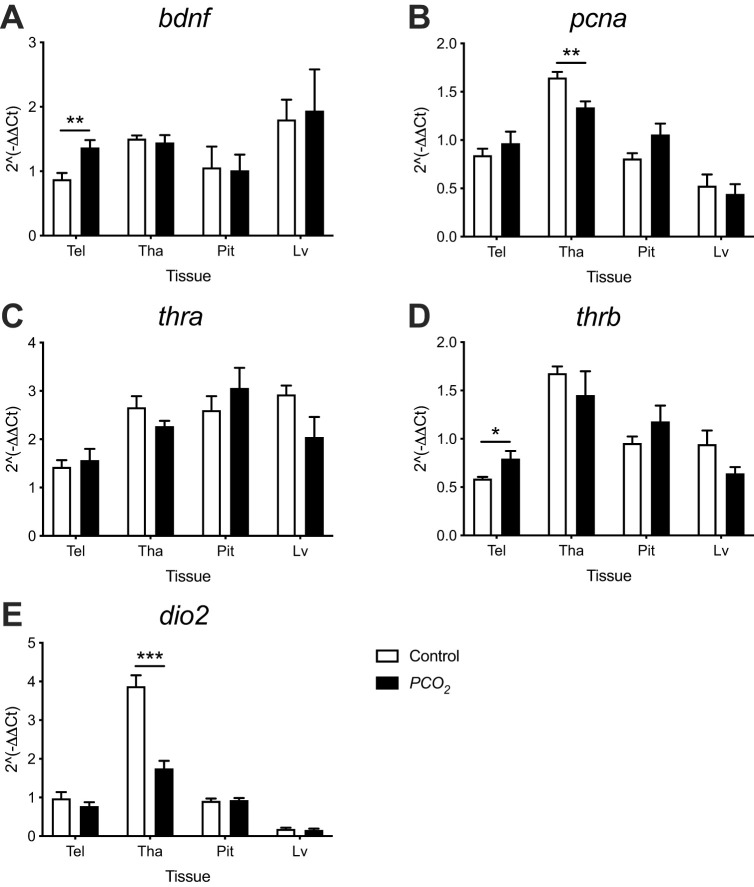
**Gene expression in the telencephalon (Tel), thalamus (Tha) pituitary (Pit) and liver (Lv) of seabream after the 4 weeks’ exposure to control (*n*=5-6) and low pH/high *P*CO_2_ (*n*=5-6) seawater conditions.** (A) *bdnf*, (B) *pcna*, (C) *thra*, (D) *thrb* and (E) *dio2*. Significant differences between target transcript expression in the control and low pH/high *P*CO_2_ exposed fish were identified using a Student′s *t*-test and are indicated with an asterisks (*P<0.05; **P<0.01; ***P<0.001). The results are shown as the mean+s.e.m.

In the telencephalon, thalamus, pituitary, and liver the expression of *bdnf*, *pcna*, *thra*, *thrb* and *dio2* was analysed after 4 weeks exposure to control or low pH/high *P*CO_2_ seawater (Fig. 6). The transcript abundance of *bdnf* (Fig. 6A) and *thrb* (Fig. 6D) were significantly increased by 56% and 35% (*P*=0.0089 and *P*=0.0431, respectively) in the telencephalon of seabream exposed to low pH/high *P*CO_2_ conditions compared to the control group, but was not changed in the thalamus, pituitary or liver. The expression of *pcna* and *dio2* was significantly decreased by 19% and 55% (*P*=0.0059 and *P*=0.0006, respectively) in the thalamus of high *P*CO_2_-treated fish compared to the control group after the 4 weeks’ exposure (Fig. 6B and E, respectively), but was not significantly changed in the telencephalon, pituitary or liver. The expression of *thra* did not vary significantly (*P*>0.05) between the control and treated fish in any of the four tissues analysed (Fig. 6C).

## DISCUSSION

Ocean acidification affects many physiological functions in different marine organisms; many studies have shown effects of OA on fish behaviour; however, the physiological mechanisms involved in behavioural alterations are not completely understood. Early studies on the behavioural effects of OA in fish were carried out on larvae reared under high CO_2_ conditions (Dixson et al., 2010; Rossi et al., 2018) and in adults exposed to low pH/high *P*CO_2_ water for short periods (Dixson et al., 2015), usually around 4 days since this is the duration that induces behavioural alterations (Munday et al., 2010). Such short-term studies tend to look at the dynamic response during acclimation. More recently, studies have emerged describing behavioural effects of long term (Lai et al., 2017a) and even life long and transgenerational exposures to future ocean PCO2/pH conditions (Welch et al., 2014). These long-term studies are important to establish whether fish can restore normal homeostasis or if they acquire a new homeostatic state. We have previously shown that medium term exposure of seabream to low pH/high *P*CO_2_ water induces morphological changes in the OE (Velez et al., 2019); here, we show that the effects of OA start shortly after exposure and continue progressively across the time of exposure. An increase in the number of mucus cells (Fig. 3A) and a decrease in the sensory epithelium area (Fig. 3B) were seen from 1 week onwards, becoming statistically significant at 4 weeks. These observations demonstrate that changes in the OE start shortly after exposure to low pH/high *P*CO_2_ and do not reverse with time; thus, the OE adopts a different morphology presumably to adapt to the new environmental conditions.

Based on previous studies we selected genes for analysis in the present study that were differently expressed in fish exposed to OA conditions. The *cacna2d* is a voltage-dependent calcium channel (Calcium Voltage-Gated Channel Auxiliary Subunit alpha 2/delta 2) that is involved in neuronal excitability (Barclay et al., 2001; Edvardson et al., 2013), which is downregulated after 7 days' exposure to future oceanic *P*CO_2_ conditions in the olfactory epithelium of sea bass (Porteus et al., 2018). The *g-olf* is involved in olfactory transduction, a process initiated by the binding of an odorant to the olfactory receptor, which then activates an olfactory-specific G protein (G-olf). Transcription of *g-olf* was downregulated in the black seabream, *Acanthopagrus achlegelli*, exposed to high CO_2_ water for 2 weeks (Jiahuan et al., 2018). Similarly *gria4a* (glutamate receptor, ionotropic, AMPA4a) a ligand-gated ion channel involved in synaptic plasticity (Hoppmann et al., 2008; Sheng and Lee, 2001) was downregulated in the brain of sea bass after 7 days' exposure (Porteus et al., 2018). Considering previously described OA driven changes in gene expression and changes in OE morphology, we expected to find changes in the expression of the selected genes after 4 weeks' exposure to low pH/high *P*CO_2_ water. However, no differences in expression of *cacna2d*, *g-olf*, *gria4a*, *ompa* or *pcna* were found.

In the olfactory bulbs, and in contrast to other species (Porteus et al., 2018), the expression of genes related to GABAergic innervation (*gabra1* and *gabbr2*) were unchanged in the seabream after 4 weeks' exposure to low pH/high *P*CO_2_ conditions. As in the OE, the expression of the other genes analysed in the OB, *gria4a*, *pcna*, *kcnn3*, *grm4*, were unchanged*.* This contrasts with a study on sea bass in which *kcnn3* (a potassium channel activated by calcium and involved in decreasing neuronal excitability) and *grm4* (a metabotropic glutamate receptor), were upregulated in the OB of fish exposed to low pH/high *P*CO_2_ conditions up to 7 days (Porteus et al., 2018). But is in alignment with studies of the brain of sticklebacks, where the expression of genes involved in GABAergic neurotransmission were only slightly modified after 43 days' exposure to near-future CO_2_ conditions (Lai et al., 2017a). Taken together, the evidence suggests that some physiological responses to OA are highly species-specific and that, in seabream, the olfactory system seems to change soon after exposure.

OA conditions caused an increase in the expression of *bdnf* in the telencephalon, and a decrease in *pcna* in the thalamus. Both genes are involved in the proliferation, differentiation and survival of undifferentiated neurons (Lai et al., 2017b). In teleosts, *pcna* is a proliferation marker used to analyse neurogenic zones within the brain (Grandel et al., 2006), and the mobilization of *bdnf* in the brain promotes neurogenesis, cell survival and synaptic plasticity (Johansen et al., 2012; Mes et al., 2018). Of note is the area-specific response in the brain of seabream exposed to OA. THs are important regulatory factors of *bdnf*, and severe hypothyroidism in the early neonatal period of rodents results in developmental and cognitive impairment and reductions in the mRNA and protein expression of *bdnf* (Lasley and Gilbert, 2011). Furthermore, TH administration increases the expression of *bdnf* in the cerebellum, cortex and hippocampus (Camboni et al., 2003; Sui and Li, 2010). The increased expression of *bdnf* in seabream exposed to OA may be linked to the increased expression of the thyroid nuclear receptor type β (*thrb*), given that this receptor acts as a transcription factor that mediates TH actions (Lazcano and Orozco, 2018). In line with this idea was the reduction in deiodinase type 2 (*dio2*) expression in the thalamus of OA-exposed fish, suggesting lower conversion of T4 into T3 (Campinho et al., 2010; Orozco and Valverde-R, 2005). The reduction in transcription of *dio2* in the thalamus may be related to the decreased expression of *pcna*, and the possible disruption of neural plasticity. A role for THs in the regulation of *pcna* comes from studies of hypothyroid rats (Bunker et al., 2017; Tousson et al., 2011) and in *Xenopus laevis* tadpoles (Thompson, 2000). The change in expression of TH-signalling genes in the brain but not in liver suggests that OA had a local and tissue-specific effect on genes of the TH system. The involvement of THs in the change in gene transcripts linked to neural plasticity in response to OA highlights an area for future research.

The underlying mechanism of the behavioural changes after exposure to future OA conditions was proposed to derive from disruption in the distribution of Cl^−^ and HCO_3_^−^ ions over neuronal membranes, changing GABAergic innervation from hyperpolarizing to depolarising (Nilsson et al., 2012). For this to happen, changes in ions concentrations must occur; we have monitored the ionic homeostasis of fish by assessing plasma and EDF ion composition. We identified an immediate decrease in Ca^2+^, K^+^ and Mg^2+^ in the plasma of fish exposed to OA. After 1 week exposure, these differences were less evident and, after 4 weeks, only Mg^2+^ was significantly lower in OA-exposed fish (Fig. 1). These results suggest that, after an initial change in plasma ionic content, it is compensated through physiological adjustments in ion homeostasis. Indeed, seabream exposed to elevated CO_2_ for three months were able to maintain plasma [Na^+^], [Cl^−^] and osmolality, and this balance was partially achieved by increased rates of HCO_3_^−^ secretion in the intestine (Gregório et al., 2019); the seabream is a marine fish that adapts to the brackish water of estuaries where pH/CO_2_ fluctuations may be greater than those that are predicted with climate change (Landschützer et al., 2018). THs have osmoregulatory actions in various fish species (Deal and Volkoff, 2020) and in the gilthead seabream, exposure to low salinity increases T4 levels (Klaren et al., 2007). Thus, the observed changes in the thyroid system genes many also be related to maintenance of osmoregulation in low pH/CO_2_ exposed fish, although this hypothesis needs to be further explored. The functional consequences of the changes in [Cl^−^] are difficult to foresee since intracellular concentrations were not determined.

## Conclusions

In the olfactory system, OA-induced changes in gene expression were relatively rapid, but returned to normal whereas, within the CNS, changes were more persistent. It remains to be established if such changes are adaptive or maladaptive. The modified expression in OA exposed fish of genes regulating neuroplasticity and the TH system may be one of the mechanisms involved in this phenomenon. Further studies are required to evaluate the importance of THs in the regulation of physiological responses to high CO_2_ water. Furthermore, the effects of OA on neuromodulation are understudied and neurogenesis-related factors should receive more attention in future studies.

## MATERIALS AND METHODS

### Fish maintenance and experiments

Animal maintenance and experimentation were carried out in certified experimental facilities and followed Portuguese national legislation (DL 113/2013) under a ‘group-1’ license from the Veterinary General Directorate, Ministry of Agriculture, Rural Development and Fisheries of Portugal. The behaviour and health of all animals was monitored daily, and no evidence of infection or mortality was observed during the experiments. Gilthead seabream (*Sparus aurata*) were obtained from a commercial supplier (Maresa – Mariscos de Esteros, SA, Huelva, Spain) and maintained in the experimental station of Ramalhete (CCMAR, Universidade do Algarve). Fish were held in 1000 l flow through tanks in an open circuit supplied with oxygenated seawater, under natural photoperiod and temperature, and fed with commercial pellets (2% body weight; Sparos, Olhão, Portugal). For experiments, adult seabream (average weight 266.5±23.55 g) were randomly distributed between 12 100 l tanks (*n*=3 fish/tank) in an open circuit system kept at natural temperature and photoperiod as previously described (Velez et al., 2019). Briefly, six tanks were maintained under control conditions (pH 8.2, ∼400 µatm), while the other six were maintained at low pH/high *P*CO_2_ (pH 7.7, ∼1400 µatm) by bubbling CO_2_ gas into the water. The experiments were run for 4 weeks as previous studies have shown seabream have reduced olfactory sensitivity at this time (Velez et al., 2019).

Fish from both experimental groups (*n*=6/treatment) were anaesthetized with 2-phenoxyethanol (1:10,000, Sigma-Aldrich) in control or low pH/high *P*CO_2_ seawater. The weight (g) and length (cm) of fish was measured and blood was rapidly collected using a heparinized 1 ml syringe and immediately centrifuged for 10 min at 5000 rpm. Plasma was collected into a clean vial and stored at −20°C until use. After blood collection, fish were quickly euthanized by decapitation. A hole was opened in the skull and the EDF was collected from between the skull and meninx using a 10 µl pipette. For analysis of plasma and EDF, animals were sampled after 24 h, 1 week and 4 weeks of exposure to control and low pH/high *P*CO_2_ conditions. Molecular biology analysis was carried out on fish after 4 weeks’ exposure to control or experimental conditions and the OE, olfactory bulb and three different brain regions (telencephalon, thalamus and pituitary, due to their functional relevance and ease of dissection) and liver were sampled, fixed in RNA*later*^®^ (Sigma-Aldrich) and stored at −20°C until analysis. For histological analysis, the olfactory rosettes were collected from three fish per treatment group after 1 week, 2 weeks, 3 weeks and 4 weeks of exposure to low pH/high *P*CO_2_, fixed in 4% paraformaldehyde (4% PFA) for 24 h at 4°C and washed and stored in 70% ethanol at −20°C.

### Seawater chemistry

Seawater was pumped from the ocean into two 2000 l header tanks. In one tank, seawater was aerated with ambient air (control) and in the other the water was gassed with CO_2_ to achieve the desired pH (elevated-CO_2_ treatment). The pressure of CO_2_ (*P*CO_2_) in the seawater header tanks was maintained at the target values of pH 8.19±0.01 and pH 7.7±0.01 for the control and low pH/high *P*CO_2_ treatment, respectively, using a pH probe connected to an internal controller (EXAxt PH450G, Yokogawa Iberia). Control and high CO_2_ seawater was supplied to 100 l tanks at 2 l min^−1^. The pH (Orion star A221, Thermo Fisher Scientific), temperature (Orion star A221, Thermo Fisher Scientific) and salinity (WTW, cond3310) of seawater were recorded daily in each tank. Total alkalinity in seawater samples was analysed twice a week by Gran titration (DL15 titrator, Mettler Toledo) using a certified acid titrant (0.1 M HCl, Fluka Analytical, Sigma-Aldrich). Carbonate chemistry parameters (Table 1) were calculated in CO2SYS (Pierrot et al., 2006) using the constants K1 and K2 from Mehrbach et al. (1973) refit by Dickson and Millero (1987) and Dickson (1990) for KHSO_4_.

**Table 1.**
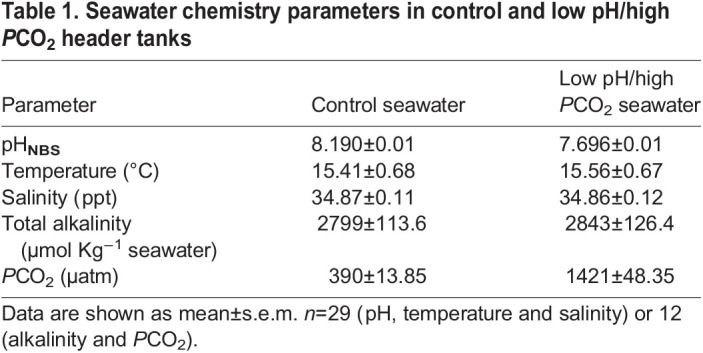
Seawater chemistry parameters in control and low pH/high *P*CO_2_ header tanks

### Ion analysis

Determination of calcium, potassium, magnesium and sodium was carried out by microwave plasma atomic emission spectrometry (MP-AES). For sample preparation, 1 µl of sample (plasma and EDF) was diluted in 999 µl of 5% nitric acid (Sigma-Aldrich), sonicated for 10 min and centrifuged at 16,000 ***g*** for 5 min at room temperature. After centrifugation, 950 µl of the supernatant was diluted in two volumes of 5% nitric acid. The ion content was analysed in an Agilent Microwave Plasma Atomic Emission Spectroscope (4200 MP-AES, Agilent Technologies). The wavelengths used for quantification was 393.336 and 396.847 nm for calcium, 766.491 nm for potassium, 285.213 and 279.553 nm for magnesium and 588.995 and 589.592 nm for sodium. The Agilent MP-AES Expert software was used to calculate the concentration based on a 12-point standard curve (range 50–10,000ppb) with automatic background subtraction provided by a blank (5% nitric acid). The limit of detection and quantification were calculated as three and ten times the standard deviation of 15 consecutive blank measurements, respectively (Li et al., 2013). Chloride quantification was carried out using a chloride thiocyanate-Hg colorimetric kit (SPINREACT S.A., Spain) and following the manufacturer's instructions; absorbance (480 nm) was quantified using a Thermo Fisher Scientific Multisken Go Microplate spectrophotometer (Thermo Fisher Scientific, Portugal).

### Histological analysis of the olfactory rosettes

To characterize the general morphology of the olfactory rosettes in control sea bream (*n*=3) and in low pH/high *P*CO_2_ (*n*=3) conditions, the tissues fixed in 4% PFA and stored in 70% ethanol were processed and embedded in paraffin. Briefly, tissue samples were dehydrated in ethanol (70%, 96% and 100%), saturated in xylene and impregnated and embedded in low melting point paraffin wax (Histosec, Merck). Serial 5 µm sections of each tissue sample were mounted on poly-L-lysine (Sigma-Aldrich) coated glass slides and stained with Masson's trichrome as previously described (Witten and Hall, 2003). Stained histological sections were observed under a microscope (Leica DM2000) coupled to a digital camera (Leica DFC480; IM50-software) linked to a computer, for digital image analysis. The software ImageJ (Abràmoff et al., 2006) was used to determine the number of mucous cells in the non-sensory epithelium as well as the ratio between the non-sensory epithelium *versus* sensory epithelium; this was obtained by dividing the length of the apical non-sensory epithelium by the total length of the olfactory lamella (from the top to the central raphe), as previously described (Velez et al., 2019).

### Analysis of gene expression by quantitative real-time PCR (RT-qPCR)

Total RNA was extracted from tissues samples (olfactory epithelium, olfactory bulb, telencephalon, thalamus, pituitary and liver) fixed in RNA*later*^®^ (Sigma-Aldrich) with an E.Z.N.A^®^ Total RNA Kit I (R6834, Omega) following the manufacturer's instructions. RNA extracts were column purified and treated with DNase using the E.Z.N.A^®^ RNase-Free DNase Set I (E1091, Omega) according to the manufacturer's instructions. RNA quality and concentration were assessed with a Nanodrop 1000 spectrophotometer (Thermo Fisher Scientific) and the integrity verified by electrophoresis on a 2% agarose gel. DNA-free total RNA (500 ng) was used for cDNA synthesis as previously described (Costa et al., 2017). Briefly, cDNA was synthetised in a 20 µl reaction volume containing 100 mM random hexamers (Specanalítica), 100 U of RevertAid Revert Transcriptase (Thermo Fisher Scientific), 8 U of NZY Ribonuclease Inhibitor (nzytech) and 100 mM of nucleotides (nzytech). The quality of the cDNA was verified by PCR amplification of 18S ribosomal RNA (18 s rRNA) with specific primers (Table 2) and the PCR products were run on a 2% agarose gel to confirm amplicon size and the absence of contamination with genomic DNA.

**Table 2.**
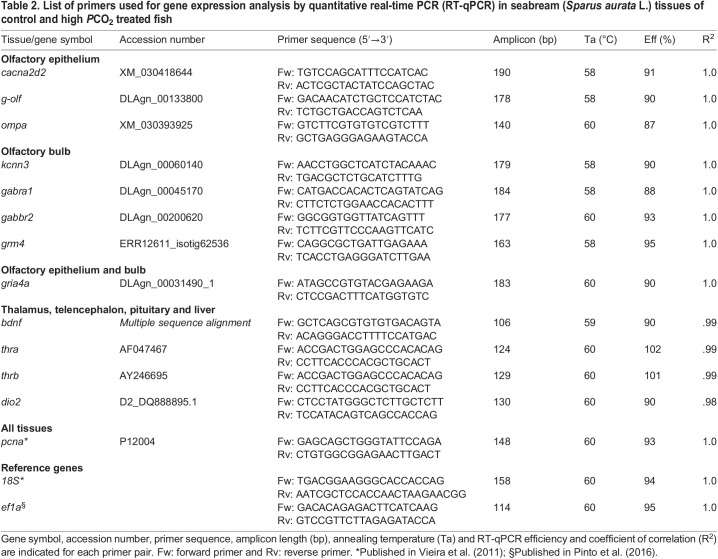
List of primers used for gene expression analysis by quantitative real-time PCR (RT-qPCR) in seabream (*Sparus aurata* L.) tissues of control and high *P*CO_2_ treated fish

RT-qPCR was used to analyse the mRNA expression of target transcripts in the tissues collected from seabream maintained in control or acidified water. Candidate genes related to excitatory neuron transmission, GABA signalling, synaptic plasticity, olfactory transduction, proliferation, neuroplasticity and the thyroid system were selected based on previous studies (Cerqueira et al., 2017; Jiahuan et al., 2018; Lai et al., 2017b; Porteus et al., 2018; Williams et al., 2019). RT-qPCR reactions were run in duplicate in 10 µl reactions containing 1x SsoFast-Evagreen Supermix (Bio-Rad), cDNA (∼16.7 ng) and 300 nM of forward and reverse primers. Specific PCR primers were designed for gilthead seabream target transcripts (Table 2) using the PrimerQuest Tool (IDT Integrated DNA Technologies, and those for the reference genes *18 s rRNA* (*18 s*) and *ef1α* have been previously reported (Table 2). Primer specificity was confirmed by sequencing PCR products and by verifying amplicon size on 2% (w/v) agarose gels. Transcripts were quantified in a StepOnePlus thermocycler (Applied Biosystems) using the standard-curve method (software StepOne™ Real Time) as previously described (Costa et al., 2017). The standard curve was generated using serial dilutions of specific PCR products for each gene (obtained using the same species, tissues, and primers for RT-qPCR analysis). A final melting curve was performed between 60°C and 95°C and produced a single product dissociation curve for each gene. Relative expression (2^−ΔΔCT^) was estimated in the olfactory epithelium and bulb using the geometric mean of *18 s* and *ef1α*, which did not vary significantly (*P*>0.05) between samples. For the telencephalon, thalamus, pituitary, and liver only *18 s* did not vary significantly (*P*>0.05) between samples and this gene was used as the reference for determination of relative expression (2^^−ΔΔCT^).

### Statistical analysis

Differences in plasma and EDF ion concentration and in the morpho-histological analysis of the OE of control and treated fish were evaluated by two-way ANOVA followed by a Tukey multiple comparisons test. Significant differences in the relative expression of target transcripts in tissues of sea bream after 4 weeks of low pH/high *P*CO_2_ exposure were assessed using a Student’s *t*-test. All the analyses were performed in GraphPad Prism 7.0a for Mac OS X (GraphPad Software, La Jolla, CA, USA, www.graphpad.com). The significance cut-off was set at *P*<0.05 and data are presented as mean±standard error of the mean (s.e.m.), unless otherwise stated.
